# Recovery strategy of fault distribution network considering collaborative optimization of recovery and repair

**DOI:** 10.1371/journal.pone.0331390

**Published:** 2025-09-03

**Authors:** Naiwei Tu, Yibo Shi, Yuqiang Hao

**Affiliations:** Faculty of Electrical and Control Engineering, Liaoning Technical University, Huludao, China; Aalto University, FINLAND

## Abstract

For the fault recovery and emergency repair after multiple faults in the distribution network, this paper proposes a fault distribution network recovery strategy considering the collaborative optimization of recovery and emergency repair. Initially, due to the difference and uncertainty between the system load demand and the distributed generation (DG) output, a bilayer dynamic fault recovery with phase type in time scale was constructed. The upper layer considers the recovery of the distribution network during faults, optimizing network reconfiguration schemes using DG outputs predicted by deep stochastic configuration network in conjunction with time-varying load demands. The lower considers the economic effect of the loss during repair and determines the optimal fault repair sequence. Furthermore, an enhanced Nutcracker Optimization algorithm to solve the bilayer model was proposed, determining the dynamic combination of the fault reconfiguration scheme and the repair sequence. Finally, to validate this strategy, this paper conducted simulations using the IEEE 33-node system. The experimental results under multiple strategies show the feasibility and effectiveness of this paper strategy, which ensures the effective recovery of fault nodes after power failure.

## 1. Introduction

In recent years, frequent natural disasters like earthquakes and heavy rainfall have led to widespread power outages, seriously affecting the safe and stable operation of power grid systems [1–3]. The distribution network, which directly serves users, operates in a complex environment and is more vulnerable to faults. Additionally, the integration of DG in the distribution network is increasing [4,5], making it a trend to connect clean energy sources like wind and solar power [6].

Power outages caused by user-side loads can result in significant economic losses for both the power grid and users [7]. Therefore, it is crucial to restore power to the distribution network as quickly as possible after a fault [8,9].

From the perspective of overall fault recovery methods, network reconfiguration and restoration, along with fault repair, are two important means for recovering from distribution network faults. With the widespread integration of DG into the grid, some scholars have begun to explore the use of DG characteristics in conjunction with distribution network reconfiguration for fault recovery. For example [10], considered reconfiguring distribution networks to minimize network loss and achieve load balancing before and after the reconfiguration [11]. focused on restoring load levels and reducing restoration times following power outages, developing a model for an active distribution network with DG operating in island mode to address fault recovery challenges. As the types of clean DG have increased, the characteristics and uncertain impacts of wind and photovoltaic output have been examined [12–14]. [15] studied the maximum supply capacity of different types of DG for active distribution network fault recovery [16]. analyzed the optimal deployment locations for DG during the black start of the distribution network and proposed a partitioned restoration method. Additionally, other scholars have conducted in-depth research on emergency fault repair. For instance [17–19], utilized DG, mobile power sources (MPS), and other renewable energy resources, considering factors such as shortest path transportation and road transport risks during emergency repairs of faulty distribution networks. While these studies provide valuable insights for developing post-disaster restoration and emergency repair strategies, they have not addressed the coordinated optimization of both approaches [20,21]. explored the relationship between the two by incorporating resilience assessment, which dynamically integrates fault location, network reconstruction, and fault repair for effective fault recovery.

The core of power system fault recovery is its recovery algorithm [22]. In terms of distribution network reconfiguration [23], had considered multiple objectives such as network loss, economy, and power, and uses an improved SSA to dynamically reconfigure the distribution network structure [24]. had proposed SMA using parallel population computing to consider the reliability, economic indicators and environmental factors of the distribution network reconstruction method during the re-construction process. For fault repair issues [25], had used an improved ant colony algorithm to obtain an optimized repair plan that considers the factor of whether the fault path is damaged. In [26], logistics scheduling and relief personnel planning had been considered, and an improved bacterial colony chemotaxis optimization algorithm had been used to solve a two-stage emergency repair model.

In summary, as the development of new energy accelerates, the integration of low-carbon grids powered by renewable energy has become an inevitable trend. For distribution grids with DG, selecting effective strategies for grid reconstruction and emergency repair is crucial for fault recovery. This paper examines how modeling time-varying load demands and DG output affects the coordination between distribution network reconfiguration, restoration, and fault repair. The goal is to ensure efficient restoration of power to users after a fault, thereby enhancing supply reliability and improving user satisfaction.

## 2. Fault recovery issues in distribution network systems

This paper investigates the restoration process using two methods—fault recovery and emergency repair—when a distribution grid loses its upper-level power supply due to disturbances, leading to widespread outages. Considering the time-varying power demand on the user side and the uncertainty of DG output, the study aims to enhance fault recovery efficiency. By introducing temporal factors to capture the varying characteristics of different loads, this study employs DeepSCN for fitting DG output. It also considers network reconstruction and emergency repair strategies to improve load restoration during the recovery process, ensuring effective restoration of power to affected users and minimizing losses from disturbances.

### 2.1. Differences in user-side load demand

To further divide the priority of restoration of power outages due to faults with different loads over time, this paper uses two methods to form Load power outage urgency $X_{i,t}$. The first ($c_{level}$) is a grading system for classifying the importance of loads and the second ($b_{i,t}$) type is calculated according to different categories of load. The mathematical formula for Load and Power outage urgency $X_{i,t}$ are as follows:


$$X_{i,t}=\;c_{level}\cdot b_{i,t}$$ (1)

where $c_{level}$ is the load classification system, which is divided into three levels: $c_{level1}$, $c_{level2}$, and $c_{level3}$. Which indicates the degree of priority of primary, secondary, and tertiary loads, respectively. The parameter is set to 10 5 1; is the classification of the importance of power outages for load nodes during peak hours $t_{1}$, normal hours $t_{2}$, and idle hours $t_{3}$.

This paper takes several typical electricity consumption patterns, such as industrial loads, commercial loads, residential loads and government unit loads, as research objects, and considers the time span to classify the importance. As shown in Table 1.

**Table 1:** Time division of several typical loads.

Different times	Industrial loads	Commercial loads	Residential loads	Government unit loads
peak hours	08:00-12:00 17:00-21:00	10:00-19:00	11:00-13:00 19:00-22:00	9:00-12:00 14:00-17:00
normal hours	12:00-17:00 21:00-24:00	08:00-10:00 19:00-24:00	06:00-11:00 13:00-19:00 22:00-24:00	07:00-09:00 12:00-14:00 17:00-24:00
idle hours	00:00-08:00	00:00-08:00	00:00-06:00	00:00-07:00

### 2.2. Application of DeepSCN for wind and solar power output prediction

Due to the characteristics of distributed generation, such as high uncertainty and strong volatility, directly simulating using mathematical distribution formulas can lead to significant errors, making it challenging to align results with reality [27,28]. This paper employs a DeepSCN to predict the wind and solar power output connected to the distribution network.

DeepSCN is a method for incrementally constructing a neural network in a random manner. It begins with one hidden node and one hidden layer. The random assignment of weights and biases is regulated by a supervisory mechanism, and the number of hidden layers is gradually increased until the maximum limit is reached. Once the maximum number of hidden layers is set, the construction process continues until the acceptable error threshold is met. Each hidden layer node is then directly connected to the output layer, and least squares are used to calculate and update the output weights of each hidden layer node.

**Fig 1:**
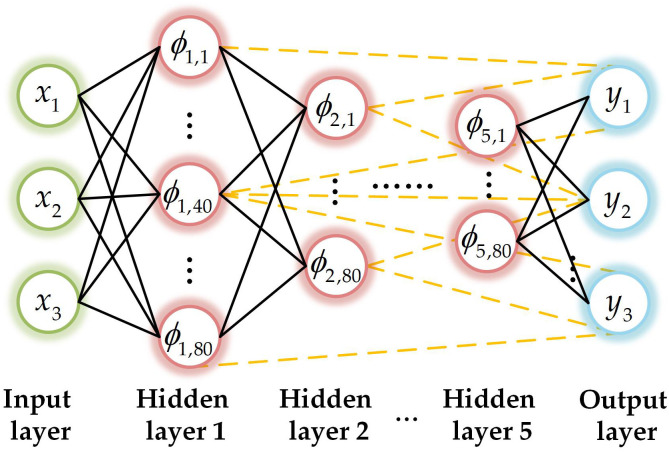
DeepSCN structure.

In this study, the maximum number of hidden layers for DeepSCN is set to 5, with a maximum of 80 hidden nodes per layer. There are 3 input nodes for predicting wind and solar output and 3 output nodes. The structure of DeepSCN is illustrated in Fig 1.

Considering the timing and complementarity of wind and solar power generation, the DeepSCN model is used to predict the data of wind and solar power generation within a day using the data of light and wind power in a region of China in 2020 as a sample. It is used to provide data support for the distributed generation output of subsequent questions, reducing errors caused by uncertainties in external data.

**Fig 2:**
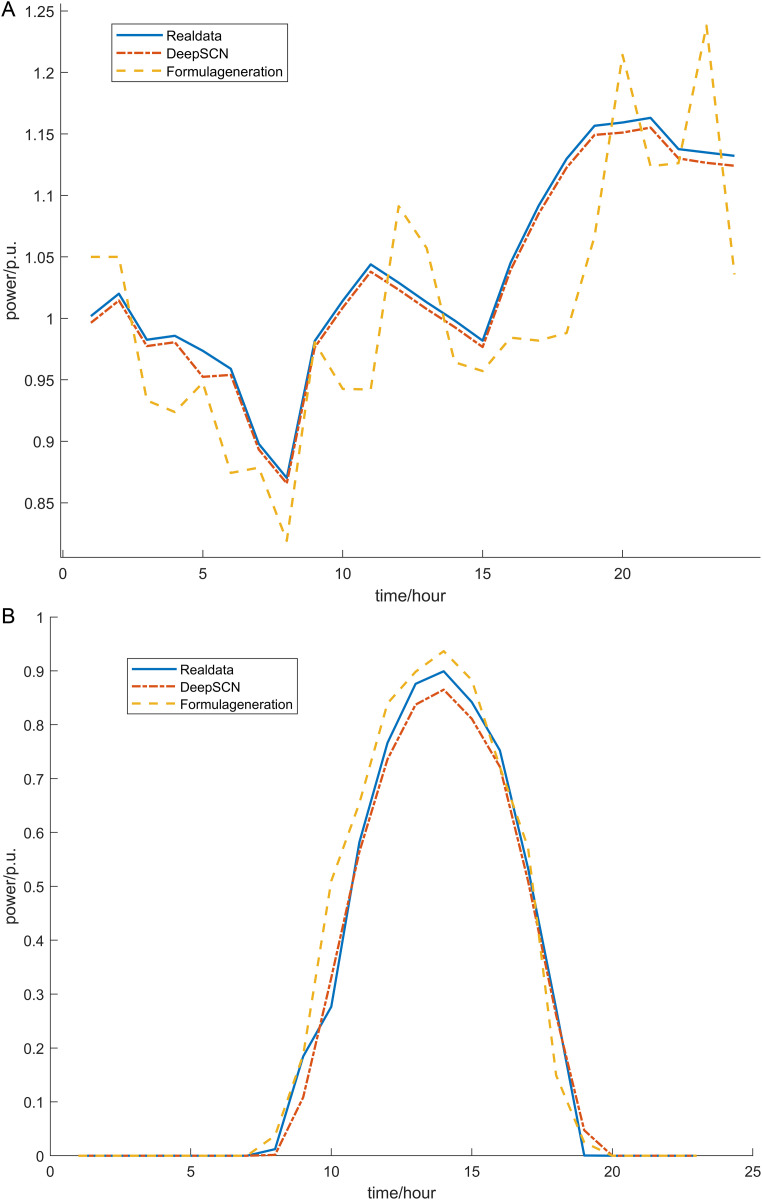
DeepSCN prediction comparison chart. (a) DeepSCN predicted wind power output. (b) DeepSCN predicted wind solar output.

**Fig 3:**
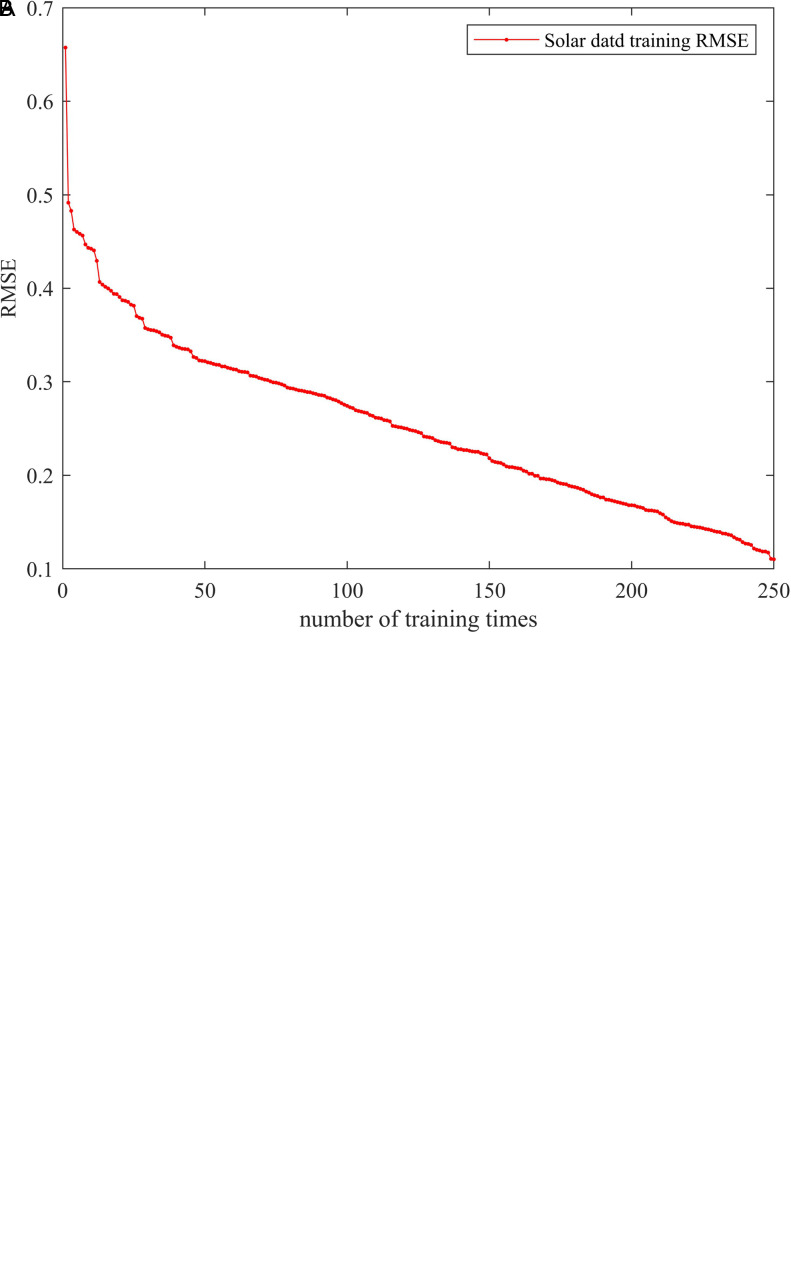
RMSE of the training results. (a) RMSE change of training wind output. (b) RMSE change of training solar output.

Simulations of wind and photovoltaic power generation models were conducted based on References [29,30]. The model formula is shown below.The parameters of photovoltaic cells and wind turbines are shown in Table 2 and 3. DeepSCN was used to predict daily wind and photovoltaic power generation data and the RMSE of the training results is shown in Figs 2 and 3.

**Table 2:** Photovoltaic cell parameters.

Solar Cell	A	η	$P_{\max}$
PV	4500 m^2^	15%	20 W/m^2^

**Table 3:** Wind turbine parameters.

Wind Turbine Set	$P_{wr}$	$v_{r}$	$v_{co}$	$v_{ci}$
Wind	500	14.5	20	3.5

Wind power output is mainly determined by wind speed at different times. The specific formula is as follows:


$$P_{W}\left(v_{x}\right)=\{\begin{array}{cc} {}*20l0 & \left(v_{x}<v_{ci}\right)\cup \left(v_{x}>v_{co}\right)\\ \frac{P_{wr}}{v_{r}-v_{ci}}\left(v_{x}-v_{ci}\right) & \left(v_{ci}\leq v_{x}\leq v_{r}\right)\\ P_{wr} & \left(v_{r}<v_{x}\leq v_{co}\right) \end{array}$$ (2)

where $P_{W}$ is the generator output power; $P_{wr}$ is the generator rated power; $v_{x}$ is the actual wind speed at the site; $v_{ci}$ is the minimum wind speed for the generator; $v_{co}$ is the maximum wind speed for the generator; $v_{r}$ is the rated wind speed for the generator.

Light intensity, the area of photovoltaic panels, and photovoltaic conversion efficiency are the main factors affecting photovoltaic power output. Assuming that the intensity of solar radiation follows a beta distribution, its probability density function can be described as:


$$f\left(P_{PV}\right)=\frac{\Gamma (\alpha +\beta )}{\Gamma (\alpha )\Gamma (\beta )}{\left(\frac{P_{PV}}{P_{\max}}\right)}^{\alpha -1}\cdot {\left(1-\frac{P_{PV}}{P_{\max}}\right)}^{\beta -1}$$ (3)

where $P_{PV}$ is the light intensity during time; $P_{\text{max}}$ is the max limit of the power output capacity of the most comprehensive PV array in a given system, and α and β is parameters from *Beta* distribution.

The approximate formula for photovoltaic power output is as follows:


$$P_{PV}=A\eta$$ (4)

where A represents the area of the plate, η represents the efficiency of the battery plate.

## 3. Consider the reconfiguration of an optimized model for coordinating recovery and emergency repair

**Fig 4:**
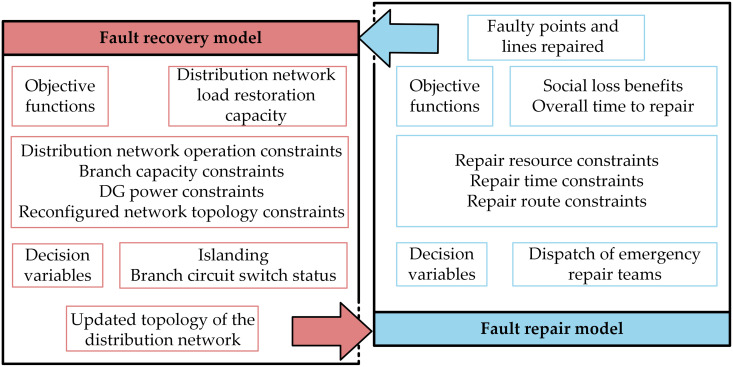
Two-stage collaborative optimization model for fault recovery.

After a fault occurs in the power grid system, during the recovery phase before the fault is resolved, DG output is first utilized as an emergency power source for small-scale islanded power supply. Subsequently, through the interconnection switches and sectionalizing switches of the power grid system, network restructuring is performed to further increase the load supply capacity and expand the scope of power. In the first stage, the fault restoration model establishes a new network structure and transmits this information to the fault repair model. The repair model then schedules maintenance personnel to address the fault points, updates the network structure, and sends the information back to the fault restoration model until all faults are repaired. Through two-stage dynamic cooperation, power failure recovery is carried out reasonably and effectively, reducing the social and economic losses caused by faults and improving user satisfaction. The collaborative optimization model of these two processes is shown in Fig 4.

### 3.1. Fault reconfiguration recovery objective function

In the recovery process following a power system failure, the primary objective is to minimize power outage loads and promptly enhance resilience performance, while considering appropriate weighting to minimize network losses and the number of switching operations. The restoration of the total lost power load serves as an indicator of resilience enhancement. The objective function is designed as follows:


$$min\;g=\int_{t_{1}}^{t_{2}}\Delta F_{res}\left(t\right)dt=\Delta F_{res}\cdot \left(J_{s,i}+J_{y,i}\right)$$ (5)

$$\Delta F_{res}=\frac{\overset{N}{\underset{i=1}{\sum \;}}\;\left|\delta_{i,t}-1\right|\cdot (X_{i,t}\cdot P_{i,t})}{\sum_{i=1}^{N}\;\left(X_{i,t}\cdot P_{i,t}\right)}$$ (6)

where N represents the total set of nodes included in the distribution network; $\Delta F_{res}$ represents the amount of system performance loss per unit time; $\delta_{i,t}$ represents the power supply status of node *i* at time *t*; $\delta_{i,t}=0/1$ represents the unpowered and powered state; $P_{i,t}$ represents the load power of node i at time t. $J_{s,i}$ and $J_{y,i}$ represent the time consumed by maintenance personnel to reach the fault point *c* and the time consumed to repair the fault line *c*, respectively.

The objective function of the overall model is as follows:


$$\min Y=w_{1}\cdot \Delta F_{res}\cdot {\left(J_{s,i}+J_{y,i}\right)}_{1}+w_{2}\cdot \sum_{l=1}^{M}\;\mu_{l,t}\cdot Z_{l}\left(\frac{P_{l}^{2}+Q_{l}^{2}}{U_{l}^{2}}\right)+w_{3}\cdot \sum_{j=1}^{N_{F}}\;(1-y_{j})+\sum_{j=1}^{N_{L}}\;{y_{j}}_{3}$$ (7)

where $w_{1}$, $w_{2}$, and $w_{3}$ represent the weights of the three considerations, which are taken as 0.7, 0.2, and 0.1 here; M indicates the total set of branches contained in the distribution network; $Z_{l}$ is the impedance on the *l*-th branch; $P_{l}^{2}$ is the active power flowing into the end of the *l*-th branch; $Q_{l}^{2}$ is the reactive power flowing into the end of the *l*-th branch; $U_{l}^{2}$ is the amplitude of the voltage at the end node of the *l*-th branch voltage amplitude; $\mu_{l,t}$ refers to the contact status of the *l*-th branch at time *t*; $\mu_{l,t}=0$, $\mu_{l,t}=1$ indicate, respectively, that *l*-th branch is disconnected at time *t* and that it is operating normally; NF is the set of sectional switches in the distribution network; NL is the set of contact switches in the current distribution network; $y_{j}$ is the opening and closing status of the *j*-th switch, $y_{j}=1$ and $y_{j}=1$ respectively represent the current closed and open status of the switch.

### 3.2. The objective function of the fault repair model

During emergency fault repair and restoration, it is important to consider the impact of the repair team’s sequence on the fault location and to determine the optimal repair route. The objective function comprehensively accounts for both the overall repair time and the economic impact of social losses. The objective function is as follows:


$$\min S=\sum_{c=1}^{N_{C}}\;(J_{s,c}+J_{y,c})+\sum_{i=1}^{N_{h}}\;\;\left[\sum_{leve=1}^{3}\;c_{level}\;\cdot P_{level,i}(J_{s,i}+J_{y,i})\right]$$ (8)

where, $N_{C}$ indicates the set of failure points; $J_{s,c}$ and $J_{y,c}$ indicate the time consumption of the repairman’s journey to the failure point *c* and the time consumption of fault repair of the fault line *c*, respectively. $N_{h}$ indicates the set of nodes representing a power failure due to a fault; $c_{level}$ indicates the hierarchical system of loads; $P_{level,i}$ indicates the demand power of different classes of loads. The $J_{s,i}$ $J_{y,i}$ respectively indicate the time consumed by the maintenance members to reach the fault point *i* and the time consumed to complete the fault repair at fault point *i*.

### 3.3. Constraints of the model

In the optimization model that considers the coordination between resilience recovery and emergency repairs, this paper takes into account various operational constraints of the power system, which are specifically described below.

1. Power flow equation constraints:

$$\begin{array}{c} P_{t,i}+P_{t,DGi}=P_{t,Li}+U_{t,i}\sum_{j=1}^{n}\;U_{t,j}(G_{ij}\cos\delta_{t,ij}+B_{ij}\sin\delta_{t,ij})\\ Q_{t,i}+Q_{t,DGi}=Q_{t,Li}+U_{t,i}\sum_{j=1}^{n}\;U_{t,j}(G_{ij}\cos\delta_{t,ij}-B_{ij}\sin\delta_{t,ij}) \end{array}$$ (9)

In the equation, *i* and *j* represent the starting node and ending node of the branch, respectively. $P_{t,i}$, $P_{t,DGi}$ and $P_{t,Li}$ respectively indicate the active power injected at node *i* at time *t*, the active power of the distributed generation at node *i*, and the active power of the load at node *i*; $Q_{t,i}$, $Q_{t,DGi}$ and $Q_{t,Li}$ respectively represent the reactive power injected by the node *i* at time *t*, the reactive power of the distributed generation at the node *i*, and the reactive power of the load at the node *i*. $U_{t,i}$ and $U_{t,j}$ respectively represent the node voltages of nodes *i* and *j* at time *t*; $G_{ij}$ and $B_{ij}$ represent the branch admittance and susceptance of nodes *i* and *j* in the distribution network structure, respectively.$\delta_{t,ij}$ is the power factor angle between nodes *i* and *j*.

1. Node current and voltage constraints:

$$\begin{array}{c} {}*20cI_{l}\leq I_{l,max}\\ U_{i,min}\leq U_{i,t}\leq U_{i,max} \end{array}$$ (10)

where $I_{l}$ and $I_{l,max}$ respectively indicate the current flowing on branch *l* and the upper limit of the current passing through branch *l*. $U_{i,min}$, $U_{i,max}$ and $U_{i,t}$ represent the lower limit of voltage, the upper limit of voltage and the voltage at *t* time of node *i* respectively;

1. ranch capacity constraints:

$$|P_{l}|\leq P_{l,m\alpha x}$$ (11)

where $P_{l}$ and $P_{l,m\alpha x}$ are the power on branch *l* and the upper limit of the power provided on branch *l*, respectively.

1. DG system power constraints:

$$P_{a,t}^{DG}={\hat{P}}_{a,t}^{DG}$$ (12)

$$Q_{a,t}^{DG}=P_{a,t}^{DG}\tan\theta_{a}^{DG}$$ (13)

$$\sqrt{{\left(P_{a,t}^{DG}\right)}^{2}+{\left(Q_{a,t}^{DG}\right)}^{2}}\leq ESS_{a,t}^{DG}$$ (14)

$$\sum_{i\in N}\;\sum_{a\in N_{k}}(P_{i,a}^{DG}-\;P_{L,i})\geq 0$$ (15)

where Nk is the total set of distribution network DG access nodes; $P_{a,t}^{DG}$ and ${\hat{P}}_{a,t}^{DG}$ respectively represent the active output and predicted active output of the a-th DG system at time *t*.$Q_{a,t}^{DG}$ indicates the reactive power output of the a-th DG at time *t*. $\theta_{a}^{DG}$ is the power factor angle of the a-th DG at time *t*.$ESS_{a,t}^{DG}$ indicates the capacity of the a-th DG at time *t*.$P_{a,t}^{DG}$ indicates the DG active power output of the access node *i*.$P_{L,i}$ represents the power required to load node *i*.

1. Network topology constraints:

g∈G (16)

where g is the network topology of the distribution network after reconstruction, and G is the set of all radial topologies in the distribution network.

6 Repair resources constraints:

Each time a repair task is performed, the repair resources of the maintenance staff are considered to be sufficient for all the repair resource consumption required for a single repair task.


$$cap_{sum}-\sum_{k=1}^{K}rep_{case(k)}\;\geq 0$$ (17)

where,$cap_{sum}$ represents the total number of emergency repair resources owned by the overall maintenance members;$rep_{case(k)}$ represents all the emergency repair resources required to complete emergency repair case; case(k) represents the sequence of fault repair points *k* to be repaired in an emergency repair case.

7 Time constraints for emergency repairs:

The maintenance staff can complete each faulty repair within the estimated repair time.


$$J_{y,k}\leq {\hat{J}}_{y,k},k=1,2,...,K$$ (18)

where $J_{y,k}$ represents the time consumed by maintenance staff in repairing the fault at point *k*;${\hat{J}}_{y,k}$ represents the estimated time consumed by maintenance staff *k* in repairing the fault; *K* represents the total number of fault points.

8 Repair route constraints:

Maintenance members can only reach and carry out fault repairs once for each fault point.


$$\sum_{\forall k=K}road_{start}^{k}=\sum_{\forall k=K}road_{k}^{final}$$ (19)

where the maintenance members start the repair case from the starting point start and return to the end point final after the case is finally completed. The starting point start and the end point final are the same. The movement route from the starting point to the *k*-th fault point for the maintenance staff to repair is described as $road_{start}^{k}$.

## 4. Cooperative optimization model solving based on an INOA

Nutcracker optimization algorithm, NOA is a new algorithm proposed by Mohamed Abdel-Basset et al. in 2023 [31], inspired by the biological habits of Clark’s nutcrackers. The authors demonstrated the effectiveness of the algorithm and its practical potential for solving constraint challenges by testing it on a test set. However, the NOA algorithm was proposed to solve continuous problems, and today’s fault recovery must deal with more than just a single continuous problem. Therefore, this paper also optimizes and improves the search strategy of the traditional NOA algorithm to make it more suitable for the requirements of this paper.

In general, the natural behavior of the star nuthatch can be understood as a global search problem with two strategies: foraging and storing before winter and searching for and retrieving stored food after winter. The original Nutcracker optimizer algorithm has problems such as the initial population not being of high quality and the search process being unstable due to the random number selection for the iterative method and transformation strategy. This paper will attempt to improve these deficiencies.

### 4.1. Improvement strategies

1. Initialize the population using the good point set and the Householder matrix refraction transformation [32].

**Fig 5:**
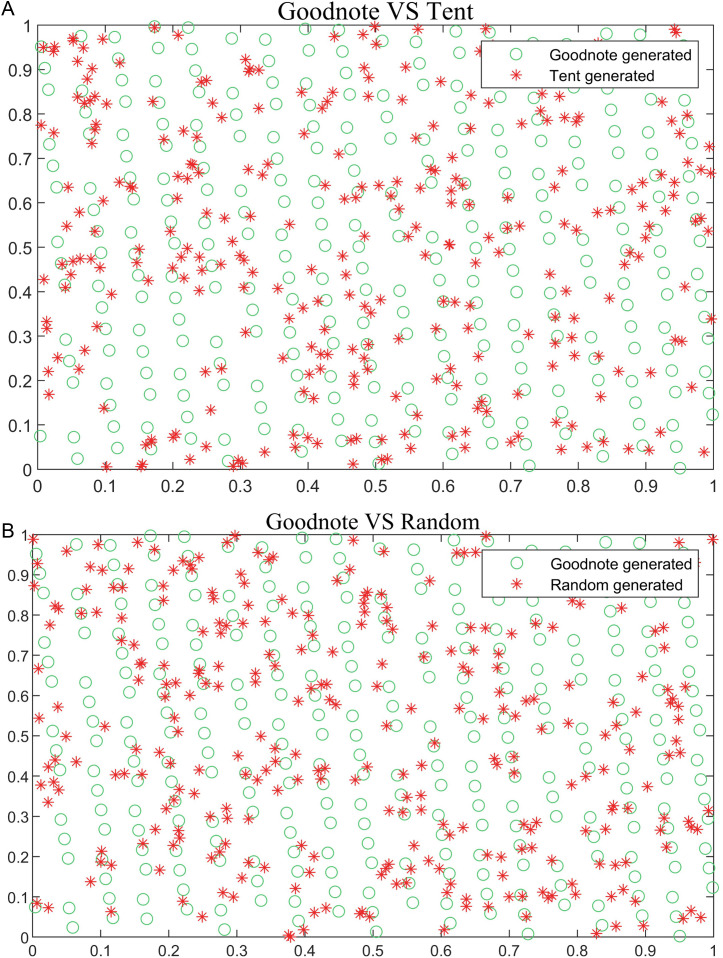
Comparison of populations generated by different methods. (a) Good point set and Tent map generate population comparison; (b) Comparison of the good point set and the randomly generated population.

The two initial populations of the original algorithm are generated randomly, which does not guarantee ergodicity or diversity. To enhance both diversity and ergodicity, population is initialized using a good point set [33] and a Householder reflection transformation. Fig 5 compare the initial populations generated using the good point set with those generated by the Tent map and random methods.

2 Adaptive parameters and transformation parameters

**Fig 6:**
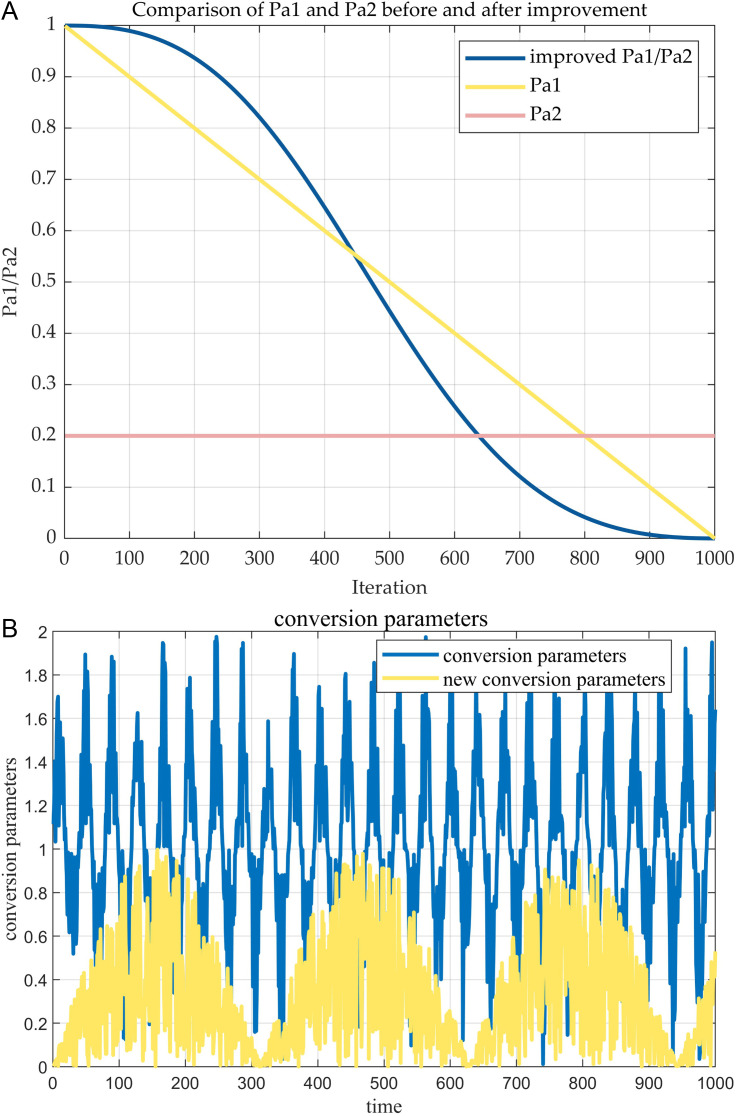
Iterative performance chart comparing before and after parameter improvements. (a) $P_{a}$CP before and after the improvement is shown in the iteration.


According to the population update formula of NOA, the development and exploration ability of the algorithm will be slowly and easily selected into the local optimum due to the change of parameter iteration in the later stage. Therefore, the iterative parameters are adaptively adjusted to improve this phenomenon. The updated parameter performance is shown in Fig 6. Here, the constant b is set to 0.01. The update formulas are as follows:

$$P_{a1}=P_{a2}=\sin(\frac{\pi \cdot t}{\;2T_{\max}}{)}^{\frac{\cos{\left(t/T_{\max}\right)}^{3}}{2}}$$ (20)

CP=|rand·sin(t*b)| (21)

3 Gold sine cosine search strategy

The use of a random number combination of two positions makes it difficult to highlight the role of the leading individual in the group. To some extent, sine and cosine is a kind of weight that can reflect this function. The golden search is combined with the sine and cosine to form a global search advantage search individual [34]. strategy (formula) before wintering is updated as follows:


$${\vec{X}}_{i}^{t+1}=\{\begin{array}{c} {}*20c\;\;\;\;X_{i,j}^{t}\;\;\;\;\;\;\;\;\;\;\;\;\;\;\;\;\;\;if\;\tau_{1}<\tau_{2}\\ \{\begin{array}{cc} {}*20cstep\cdot X_{m,j}^{t}+sin\left(r1\right)\cdot c1\cdot \gamma \cdot \left(X_{A,j}^{t}-X_{B,j}^{t}\right) & \\ +\mu \cdot \cos\left(r2\right)\cdot c2\left(r^{2}\cdot U_{j}-L_{j}\right), & t\leq T_{max}/2.0\\ step\cdot X_{C,j}^{t}+sin\left(r1\right)\cdot c1\cdot \mu \cdot \left(X_{A,j}^{t}-X_{B,j}^{t}\right) & \\ \;+\mu \cdot \cos\left(r1\right)\cdot c2\cdot \left(r^{2}\cdot U_{j}-L_{j}\right), & Otherwise \end{array}Otherwise \end{array}$$ (22)

$${X_{1}}_{i,j}^{t+1}=\{\begin{array}{c} {}*20c\;\;\;\;\;\;X_{i,j}^{t}\;\;\;\;\;\;\;\;\;\;\;\;\tau_{3}<\tau_{4}\\ \begin{array}{c} step\cdot X_{i,j}^{t}+sin\left(r1\right)\cdot c1\cdot r_{3}\cdot \left(X_{best,j}^{t}-X_{i,j}^{t}\right)\;\;\;\;\tau_{3}\geq \tau_{4}\\ \;+\cos\left(r2\right)\cdot c2\cdot r_{4}\cdot \left({\vec{RP}}_{i,1}^{t}-X_{C,j}^{t}\right) \end{array} \end{array}$$ (23)

$${X_{2}}_{i,j}^{t+1}=\{\begin{array}{c} {}*20c\;\;\;\;\;\;X_{i,j}^{t}\;\;\;\;\;\;\;\;\;\;\;\;\tau_{5}<\tau_{6}\\ \begin{array}{c} step\cdot X_{i,j}^{t}+sin\left(r1\right)\cdot c1\cdot r_{3}\cdot \left(X_{best,j}^{t}-X_{i,j}^{t}\right)\;\;\;\tau_{5}\geq \tau_{6}\\ \;+\cos\left(r2\right)\cdot c2\cdot r_{4}\cdot \left({\vec{RP}}_{i,2}^{t}-X_{C,j}^{t}\right) \end{array} \end{array}$$ (24)

where $step=gold\cdot (1+\frac{1-t}{\;T_{\max}})$.gold represents the golden ratio. sin and cos represent the sine and cosine formulas.r is a random number between 0 and 1.

4 Information share search strategy

The core idea of this approach is to allow individuals to exchange information, either directly or indirectly, using different communication operators. This promotes population diversity and leverages each other’s domain-specific knowledge. Additionally, this sharing strategy enables the algorithm to exhibit richer random behavior during the optimization process, helping to avoid getting stuck in local optima. The updated formulas are as follows:


$$x_{i}^{t+1}=\{\begin{array}{c} {}*20cx_{i}^{t}+\alpha \left(x_{j}^{t}-x_{i}^{t}⊗levy\left(\beta \right)\right)\;r<S1\\ x_{i}^{t}+\alpha \left(x_{j}^{t}-x_{i}^{t}\right)⊗levy\left(\beta \right)\;r\geq S1\\ x_{i}^{t}+\alpha \left(x_{j}^{t}⊗levy(\beta )-x_{i}^{t}\right)\;\;r\geq S2\\ x_{i}^{t}\cdot rand+x_{j}^{t}\cdot \left(1-rand\right)\;\;\;\;r\geq S3 \end{array}$$ (25)

where r is a random number satisfying uniform distribution in (0, 1);S1,S2, S3 are variables based on probability to determine which information sharing method is adopted by individuals in the population. In order to enable individuals in the population to choose each domain search method with the same probability,S1,S2,S3 are 1/4, 2/4, 3/4.

### 4.2. The performance evaluation of the INOA

**Fig 7:**
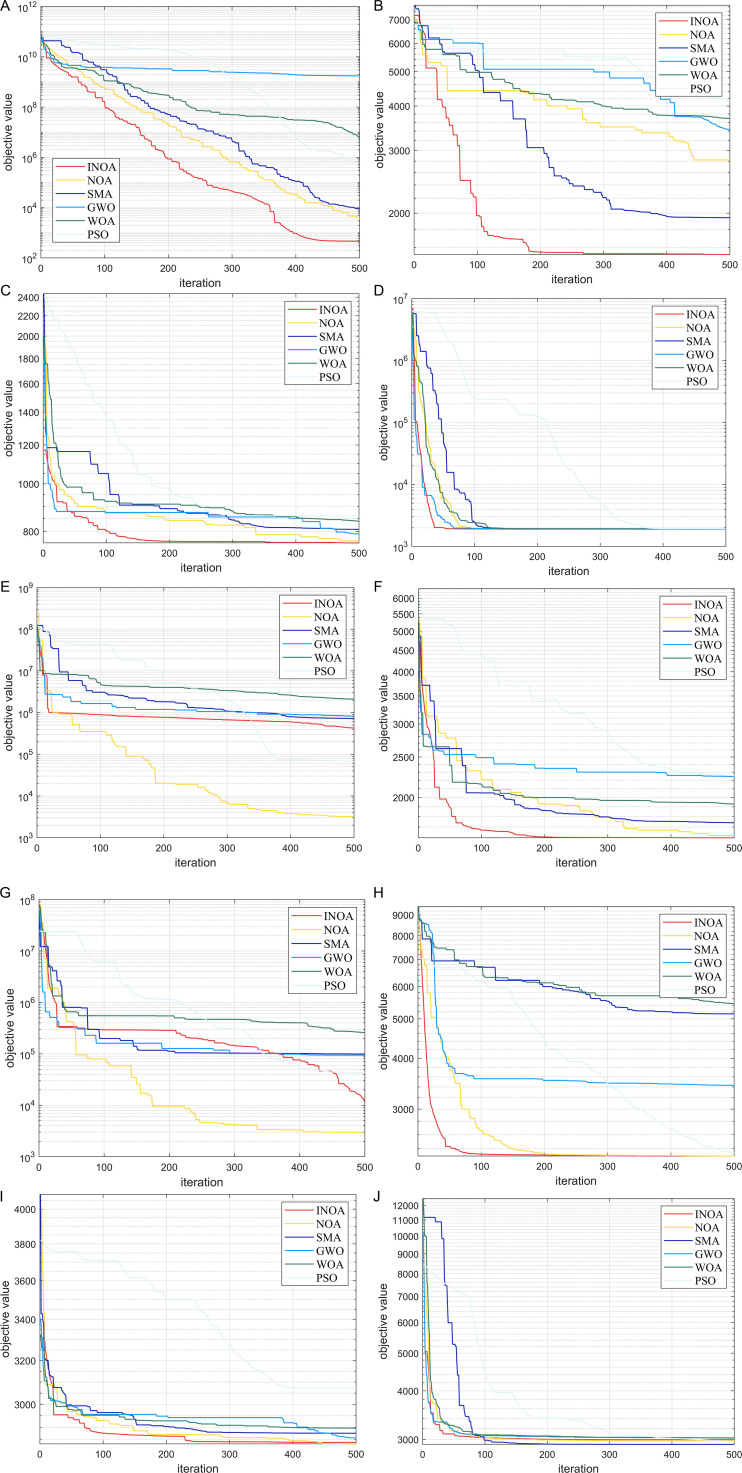
Test function performance chart. (a) Results of the F1; (b) Results of the F2; (c) Results of the F3; (d) Results of the F4; (e) Results of the F5; (f) Results of the F6; (g) Results of the 7; (h) Results of the F8; (i) Results of the F9; (j) Results of the F10.

To verify the effectiveness of the strategy used to improve NOA in section 3.1, the functions in the CEC2020 function test set are used as a reference for evaluation. It contains unimodal function, simple multimodal, mixed function and combination function. The NOA initial algorithm and four others widely used algorithms were selected for comparison: (slime mold algorithm) SMA, (gray wolf optimizer) GWO, (Whale optimization algorithm) WOA and (particle swarm optimization) PSO. The initial population size of all algorithms was set to 30, and the maximum number of iterations was set to 500. The dimension was 20. To more clearly and intuitively compare the performance of each intelligent optimization algorithm for the test function, the change curve of the fitness value of each function in the test set is shown in Fig 7. The detailed data after running 5 is shown in Table 4 below.

**Table 4:** Comparison of results from multiple runs.

function	indicator	INOA	SMA	NOA	GWO	WOA	PSO
F1	MD STD OPT	**1378.23** **1673.24** **317.358**	6771.079 9867.093 543.630	5736.335 5010.207 787.6592	8.3E + 08 3.19E + 08 5.86E + 08	348089622 6.87E + 08 1126891.562	95330849.84 1.54E + 08 877901.332
F2	MD STD OPT	**1663.632** 349.827 **1232.648**	2959.660 **183.548** 2646.569	2190.400 275.889 1725.670	3010.0897 973.127 2071.849	3145.007 358.441 2905.863	4698.564 1222.059 2744.367
F3	MD STD OPT	**752.327** **6.298** **730.338**	765.399 12.130 757.112	767.525 12.454 749.863	782.551 7.019 770.766	838.796 32.200 784.430	883.521 39.245 825.494
F4	MD STD OPT	1905.643 **0.559** 1902.874	1907.137 2.264 1904.723	**1903.809** 1.344 **1902.358**	1922.445 19.127 1907.507	1948.718 33.393 1906.921	1942.448 9.293 1930.374
F5	MD STD OPT	231027.28 100514.43 128435.66	312197.97 88850.97 188510.26	**3389.75** **412.369** **2911.90**	892688.50 684297.61 70446.377	1351209.73 708552.62 254326.742	745909.69 385560.41 357919.48
F6	MD STD OPT	**1620.507** **14.73801** **1602.140**	1628.444 49.0074 2116.007	1662.80 48.053 1603.15	1872.479 134.714 1713.415	1784.186 138.386 1671.616	2315.961 168.539 1627.855
F7	MD STD OPT	206193.44 93402.102 108632.18	513232.6 333332.3 127355.2	**2987.97** **130.855** **2756.55**	339142.55 447176.07 16605.960	313422.046 199349.063 35083.268	51111.178 80462.685 7849.7078
F8	MD STD OPT	**2300.233** **0.113339** **2300.022**	2725.928 845.5860 2301.452	2301.54 1.2816 2300.12	2418.1759 78.634782 2342.54	4376.9454 1832.0734 2319.92805	3181.7161 1389.1033 2311.444
F9	MD STD OPT	**2852.904** **12.59456** **2840.326**	2864.130 18.2229 2822.217	2856.07 15.1915 2840.89	2867.864 26.868654 2848.0025	2890.7563 27.727845 2858.3600	3199.8858 49.307118 3147.2584
F10	MD STD OPT	**2938.457** 29.8086 2914.069	2958.911 37.4353 **2914.027**	2996.316 **12.14081** 2972.53	2989.220 39.18917 2947.447	2995.14581 37.4178846 2923.97996	2966.48039 22.3440690 2930.87062

In the single-peak test function F1, the INOA algorithm has shown its ability to stably find the optimal solution. As the iterations progress, the algorithm gradually converges, while the other algorithms have not shown convergence, as they have stopped making progress after falling into a local optimum. This shows the ability of the INOA algorithm to break through local optima. For the simple multimodal test functions F2–F4, the INOA also demonstrates its excellent ability to quickly find an optimum and escape from local optima. For the remaining mixed and combined functions, INOA also exhibits good fast convergence, post-optimization, and the ability to escape from local optima. In summary, it can be proved that the INOA has strong advantages.

Based on a comprehensive analysis of the data, we can evaluate the performance of the INOA algorithm on the F1 to F10 function structures from three aspects: optimal value (OPT), standard deviation (STD), and mean value (MD).

On the F1 to F10 test functions, INOA shows outstanding performance in terms of OPT. Ranking comparisons reveal that INOA significantly outperforms other algorithms on most functions, consistently ranking among the top. This indicates its ability to approach the global optimum and showcases its exceptional optimization capabilities. Compared to algorithms such as SMA and PSO, INOA demonstrates stronger performance in handling complex optimization problems.

Regarding stability, analysis of the STD for the F1 to F10 functions shows that INOA performs exceptionally well. It has better rank in standard deviation for most functions, indicating stable and consistent results across multiple independent runs. This low volatility minimizes inconsistencies from random initialization, allowing INOA to maintain high stability across different testing environments. In contrast, algorithms like SMA and PSO typically have higher standard deviation rankings and poorer stability.

Based on the MD analysis of functions F1 to F10, INOA’s average results across multiple runs also outperform other algorithms. Comprehensive comparisons show that INOA ranks highly in average value for most functions, indicating not only high-quality solutions but also a significant advantage in convergence speed. Compared to algorithms like SMA and PSO, INOA approaches the optimal solution more quickly, demonstrating a clear advantage in optimization efficiency.

In summary, INOA’s performance on the test set highlights its significant advantages.

### 4.3. Discrete search strategy in fault recovery

#### 4.3.1. Loops network coding strategy.

In distribution network fault recovery, network reconfiguration involves changing the branch switches between nodes and the connection switches to optimize the topology of the distribution network. Due to the radial constraints of distribution network operation.

The individual encoding length under the loop-based strategy equals the number of switches in the loops. During encoding, each switch within a loop is assigned a unique natural number, and switches within a single loop are encoded only once. Thus, this paper employs a decimal-based coding strategy based on loops.

**Fig 8:**
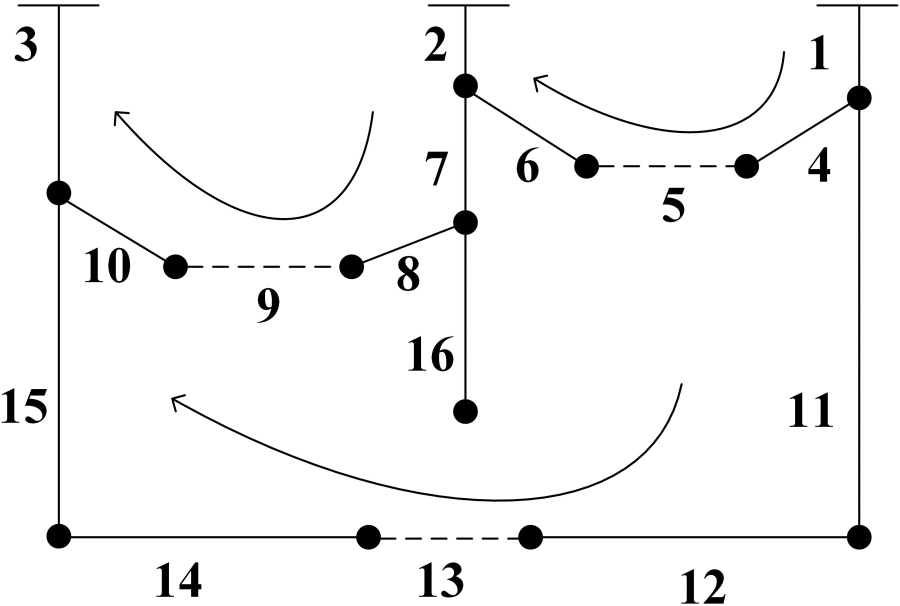
16-node network structure diagram.

For example, consider the system shown in Fig 8, a network diagram with 16 nodes. According to binary encoding rules, the encoding length of an individual is 16, resulting in a search space of 2^16 ^=65536. The loop-based encoding strategy is more efficient, with a search space size of 5·6·7=210. Based on graph theory, it can be calculated that there are 190 feasible solutions. This approach yields effective candidate solutions that account for 190/210 = 90.47% of the overall search space. The specific encoding is shown in Table 5.

**Table 5:** 16-node loop network encoding results.

Loop number	Loop branch number participating in encoding	Corresponding ring code number
1	1, 2, 4, 5, 6	1, 2, 3, 4, 5
2	2, 3, 7, 8, 9, 10	1, 2, 3, 4, 5, 6
3	1, 3, 11, 12, 13, 14, 15	1, 2, 3, 4, 5, 6, 7

#### 4.3.2. Discretization of decision variables.

For distribution network fault recovery, the decision variables are the two states of the switches between nodes, and in the INOA, the iterative position update of the Nutcracker swarm is continuous. The updated location of the star cluster needs to be discretized.

The formula is as follows:


$$X_{i}^{t+1}=\{\begin{array}{c} {}*20c1,\;r>S(X_{i}^{t})\\ 0,\;r\leq S(X_{i}^{t}) \end{array}$$ (26)

$$S(X_{i}^{t})=\frac{1}{\;1+\exp(X_{i}^{t})}$$ (27)

where r is a random number in the range 0 and 1;S(x) is the S-type discrete function.

For fault repair, the decision variable is the order of repair for each fault. As above, the location of the star cluster needs to be encoded in decimal, and the updated location of the star cluster needs to satisfy the constraint of being a positive integer. The formula is as follows:


$$X_{i}^{t+1}=\;\;round\;\;(\;X_{i}^{t})$$ (28)

where round(X) is the rounding function.

### 4.4. Co-optimization model solving process

**Fig 9:**
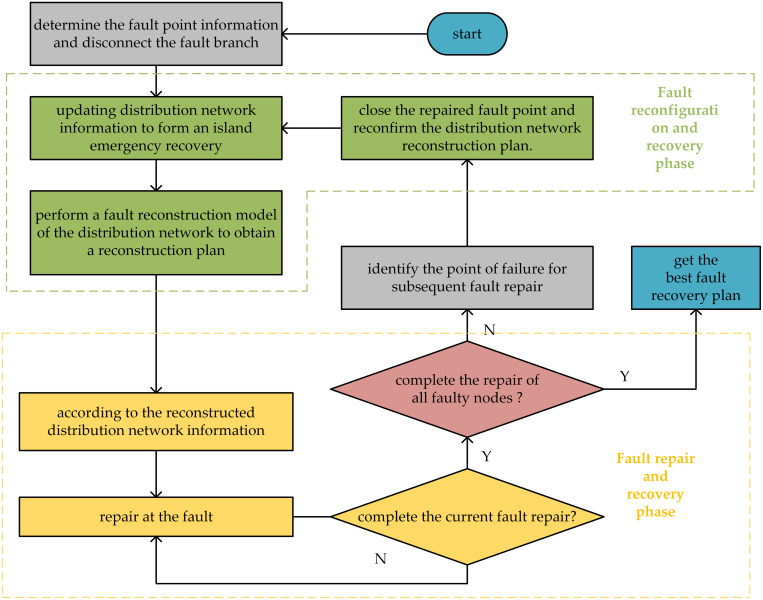
Distribution network fault emergency recovery and fault repair collaborative recovery flow chart.

In actual emergency repair operations, numerous factors interact with one another. The goal is to ensure that the system quickly regains its resilience and returns to normal operation while minimizing economic losses. The specific process flow is illustrated in Fig 9. The specific process is as follows.

**Fig 10:**
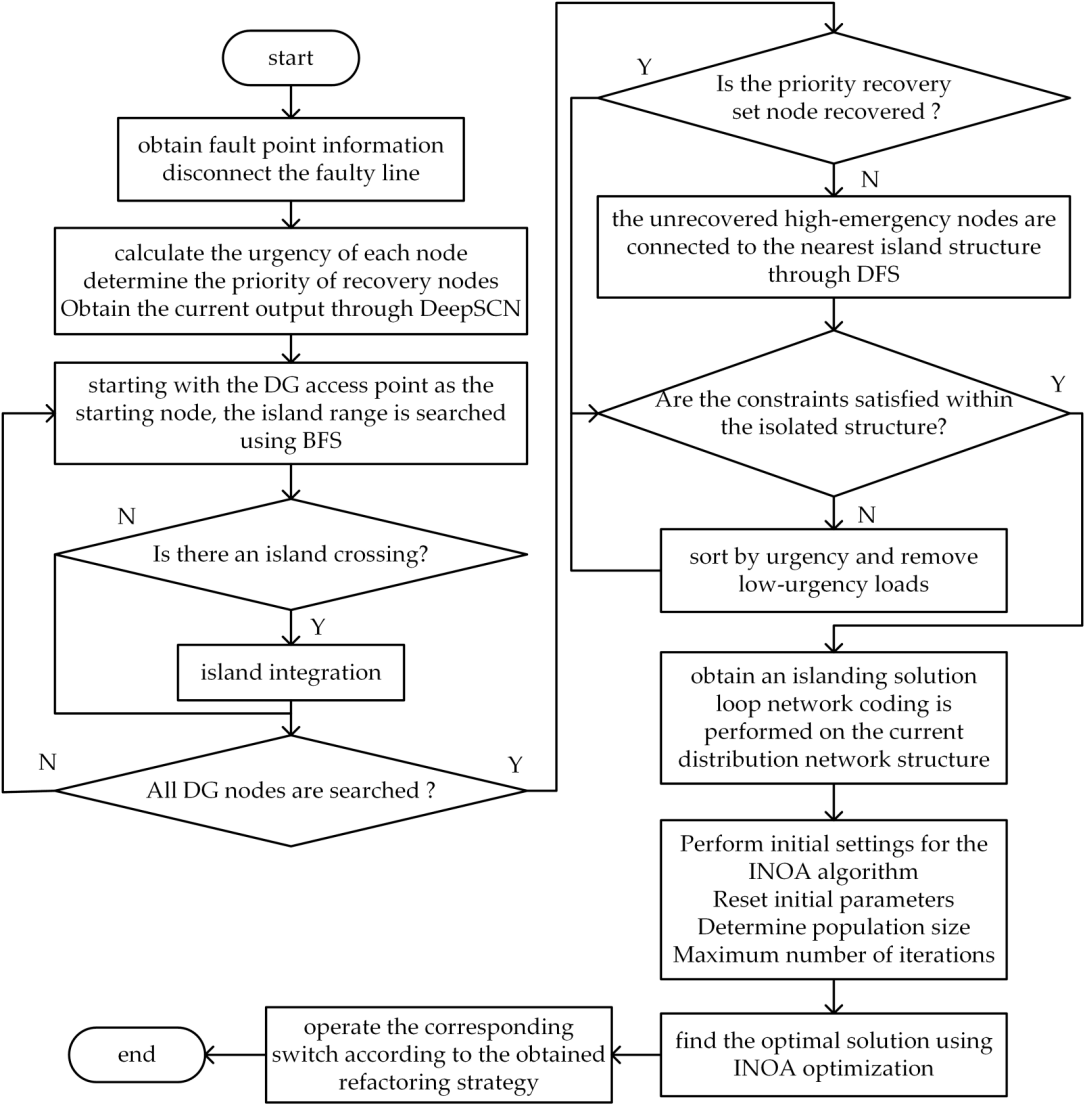
Fault reconfiguration and recovery phase flowchart.

First, determine the distribution network structure and current fault information. Calculate the priority restoration coefficient for each load and obtain the current output status of the DG. Initialize the INOA algorithm and perform the distribution network reconfiguration for resilience restoration. The specific flowchart is shown in Fig 10.

**Fig 11:**
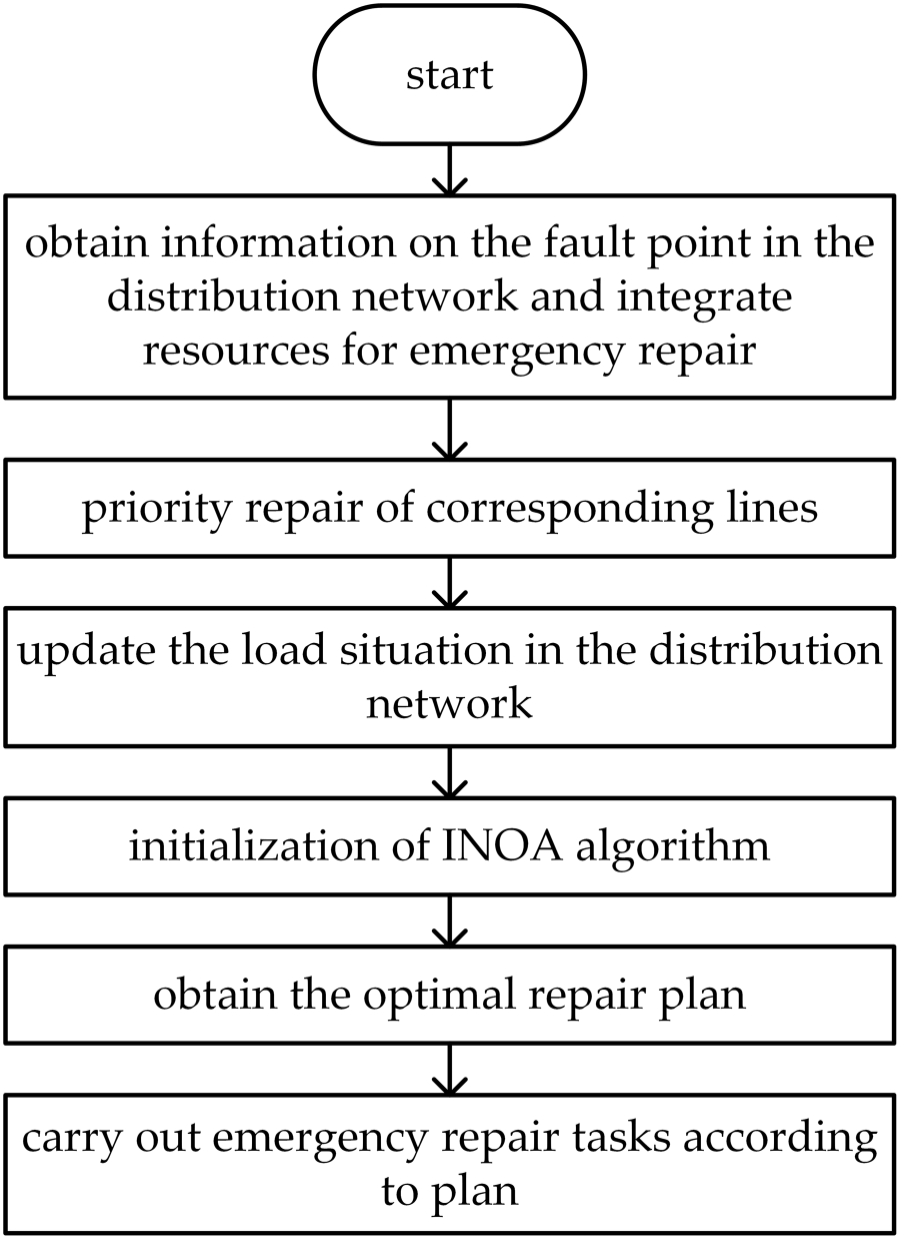
The fault repair and recovery phase flowchart.

Dispatch and allocate repair teams based on the distribution network and fault information. Initialize the INOA algorithm to determine the repair sequence for multiple faults. Assume that the repair times for all fault points are known before the repair personnel start, they have sufficient materials for all tasks, and all fault nodes can be successfully repaired. The specific flowchart is shown in Fig 11.

## 5. Results and discussion

### 5.1. Experiment on the reconfiguration of distribution networks considering load variation

**Fig 12:**
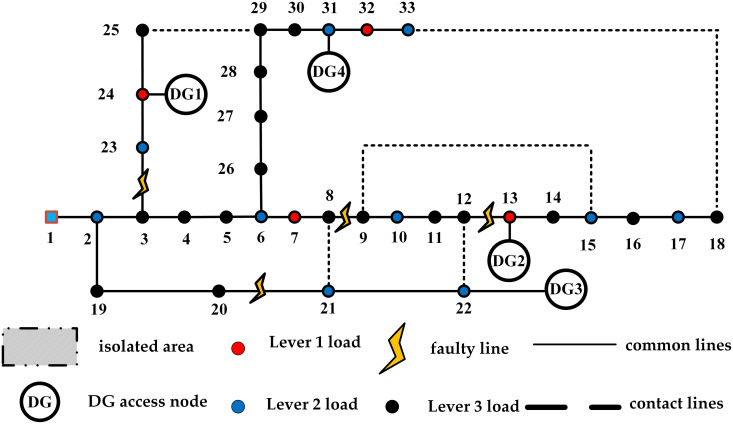
Reconstruct the experimental distribution network structure diagram.

To further verify the feasibility of the proposed distribution network fault emergency recovery strategy, three fault points have been randomly generated along the distribution network lines. The evaluation takes place during two typical time periods: 9 AM and 6 PM, allowing for the consideration of dynamic factors affecting the emergency recovery process. The structure of the distribution network system is illustrated in Fig 12, where it is noted that the contact switches in the distribution network are open by default. The output and load requirements of the DG are based on the instantaneous values at the time of failure. Specific information is provided in Table 6. The following two strategies are adopted for experimental comparison. The goal is to optimize the restoration of power to affected loads while maintaining system stability and minimizing downtime.

**Table 6:** Reconfiguration of DG system parameters in the experiment.

Power supply	Access node	Period 1 output active power/KW	Period 2 output active power/KW
DG1	24	300	500
DG2	13	410	450
DG3	22	300	450
DG4	31	280	500

Strategy 1: The emergency recovery strategy proposed in this paper is carried out for the distribution network system after the fault, and the reconfiguration scheme of the island and the main network is carried out.

Strategy 2: After the fault, the distribution network is divided into islands, and the remaining power loss load is restored by the main network.

**Fig 13:**
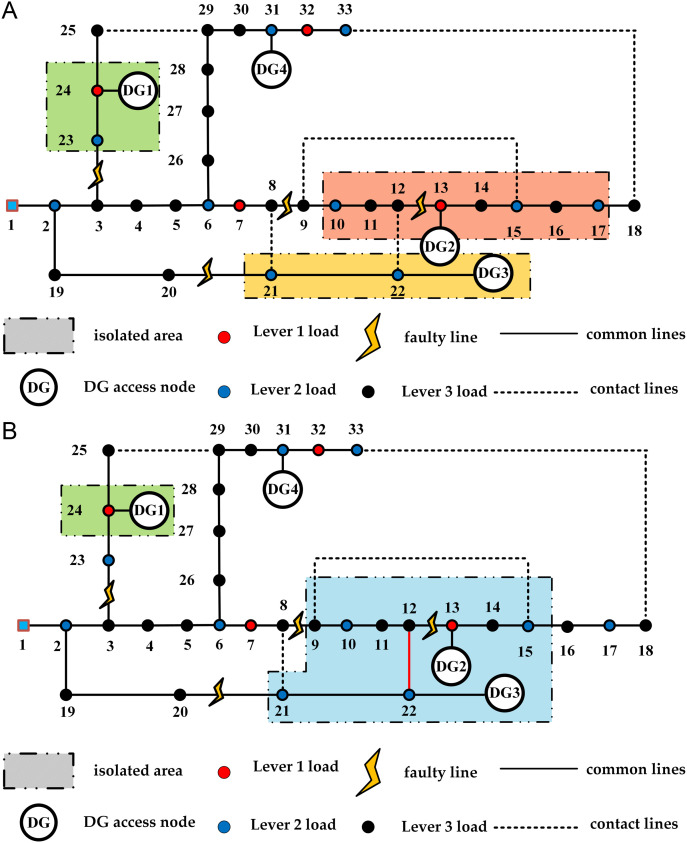
The island division results for the two periods. (a) Results of the period 1; (b) Results of the period 2.

**Fig 14:**
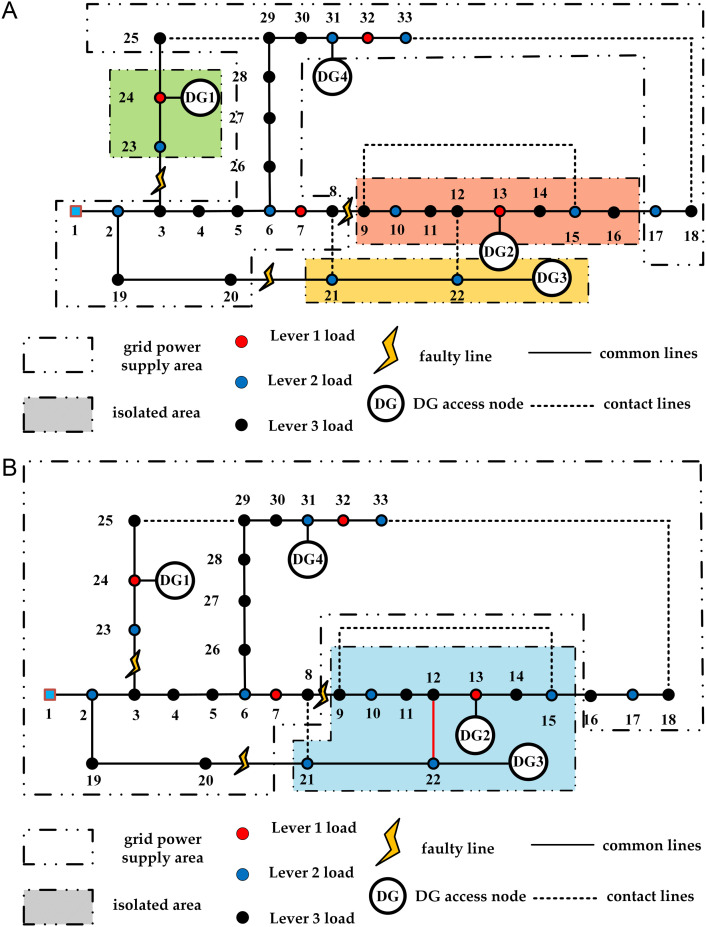
Strategy 1 two-period distribution network results diagram. (a) Result of strategy 1 in period 1; (b) Result of strategy 1 in period 2.

**Fig 15:**
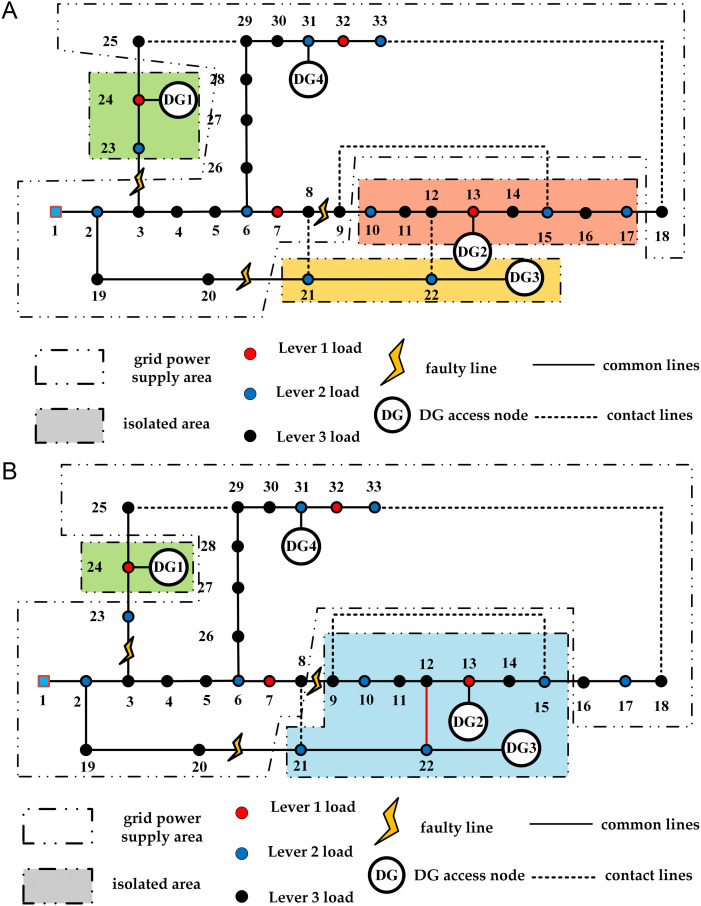
Strategy 2 two-period distribution network results diagram. (a) Result of strategy 2 in period 1; (b) Result of strategy 2 in period 2.

The island division results of the two periods are shown in Fig 13. The distribution network reconfiguration schemes of strategy 1 and strategy 2 are shown in Figs 14 and 15 respectively. The experimental results of the two strategies are shown in Table 7.

**Table 7:** Comparison of the results of the strategies run in the emergency recovery phase.

Failure period	Strategy	Loses load	Network losses/KV	Number of operations
9:00-10:00 (period 1)	strategy1	0	181.30	2
	strategy2	9	178.28	2
18:00-19:00 (period 2)	strategy1	0	448.72	3
	strategy2	23	407.05	3

The experiment shows that Strategy 1 results in a slight increase in network loss compared to Strategy 2. However, in the recovery plan for period 1 under Strategy 1, no nodes experience power outages due to failures, while in Strategy 2, node 9 suffers a power outage due to insufficient DG power generation. Similarly, in period 2, Strategy 1 ensures the power supply for node 23, avoiding power outage losses in the load nodes and maximizing the reconfiguration objective function.

In the initial island division structure, failures occur at different times, leading to time-varying changes in the wind and solar power DG connected to the system and load demand. This results in changes to the island division structure during the two time periods of failure. In period 2, the DG2 and DG3 systems merge into a larger island through the contact line, maximizing the recovery range of the island.

In period 1, the DG2 system initially included node 9 but not node 17. However, since the load urgency of nodes 16 and 17 was greater than that of node 9, the lower urgency load of node 9 was reduced, allowing node 17 to be restored first while node 9 was abandoned. In period 2, due to the high load on the user side, the power generation from the DG2 and DG3 systems was insufficient to restore node 17, ensuring reasonable restoration on the user side with effective resources.

From the above, it is clear that after a multi-point failure, simply dividing the island and reconfiguring the distribution network is insufficient for effective load restoration. It is also necessary to reconfigure the distribution network lines and utilize the main grid for load restoration.

### 5.2. Coordinated fault recovery strategy for distribution networks

#### 5.2.1. Case settings.

This paper uses the IEEE 33-node distribution network system as a case study for simulation. The objective is to validate the effectiveness of the fault distribution network restoration strategy proposed herein, which involves coordinated optimization of restoration and repair efforts. Under extreme natural disaster conditions, it is assumed that the distribution network system experiences large-scale disturbances, leading to multiple faults occurring simultaneously. Eight fault points are established, taking into account various fault types and geographical locations.

**Fig 16:**
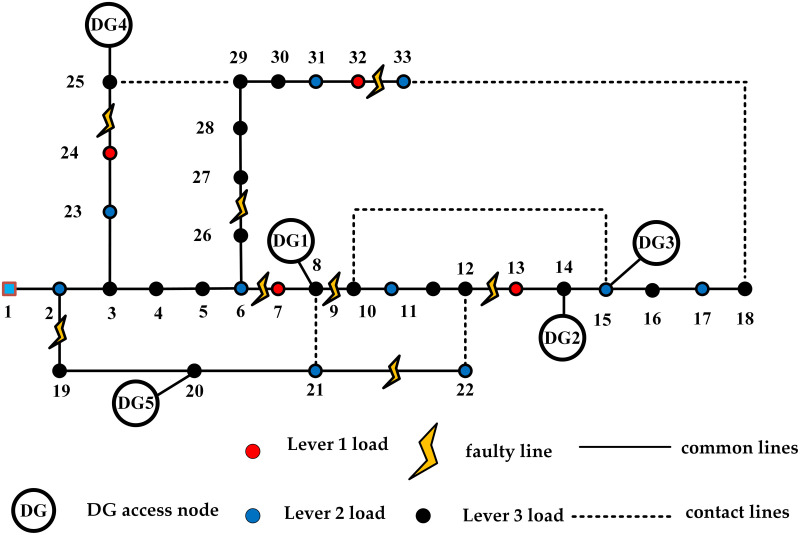
The structure of distribution network in collaborative strategy experiment.

The IEEE 33-node system operates with a reference voltage of 12.66 kV, and node 1 is designated as the root node. The primary load nodes are defined as nodes 7, 13, 24, and 32; secondary load nodes include nodes 2, 6, 10, 15, 17, 21, 22, 23, 31, and 33; while the remaining nodes are designated as tertiary load nodes. Industrial load nodes are identified as 8, 24, 25, and 30. Commercial load nodes consist of 2, 4, 11, 15, 17, 24, 27, and 33. Residential load nodes include 3, 5, 6, 9, 10, 12, 14, 16, 18, 19, 20, 21, 23, 26, 28, 29, and 31. Institutional load nodes are 7, 13, and 32. The DG connection nodes are 8, 14, 15, 20, and 25. The system structure diagram of this distribution network is illustrated in Fig 16, with the contactors shown in their default open state.

An emergency repair team is formed to initiate repair operations starting from fault point C8, traveling at a speed of 20 km/h. Only the straight-line distance between two points is considered for fault distance calculations. The types of the eight fault points, estimated repair times, and geographic coordinates are summarized in Table 8 (Fault Point Details). In the event of a fault occurring at 10:00, both the output of the DG and load demand are taken as the instantaneous values at the time of the fault.

**Table 8:** Fault point details.

Fault point	Failure mode	Repair time	Fault coordinates
C1	10kV line power failure	1.2h	(114.043951, 25.073216)
C2	Line switch failure	0.4h	(114.015777, 25.035073)
C3	Damage to transformer body	0.5h	(114.121448, 25.077283)
C4	Damage to 10kV ring network cable head	0.8h	(114.077036, 25.086924)
C5	Cable damage	1.2h	(114.073303, 25.124912)
C6	Grounding damage for dual power supply users	0.8h	(114.078628, 25.141267)
C7	Overhead line and switchgear failure	1.0h	(114.098981, 25.136718)
C8	Damage to 10kV ring network cabinet vacuum switch	0.7h	(114.034902, 25.133250)

To verify the effectiveness of the proposed strategy, three scenarios are set up.

The first case does not consider load recovery volume and repair scheduling. The repair team randomly repairs all fault points, and after completion, implements a fault resilience recovery strategy.

The second case does not consider load recovery volume. The repair process only considers the shortest repair time and repair distance for a coordinated fault recovery strategy.

The third case considers fault recovery and repair coordination recovery strategies during the repair process.

#### 5.2.2. Consider an optimized plan for restoring and repairing coordination.

**Fig 17:**
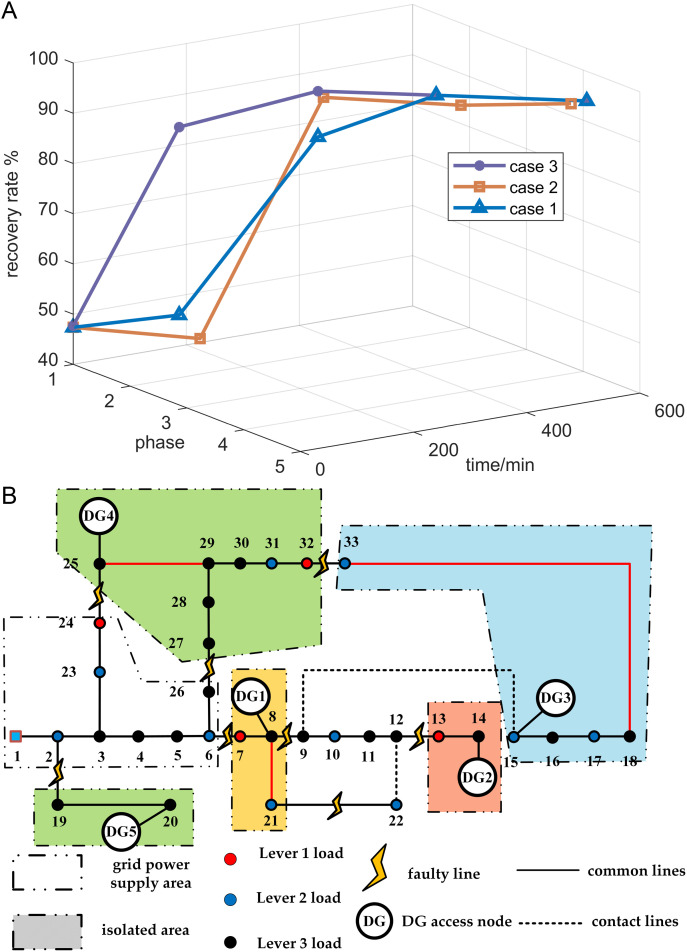
Recovery rate changes for the three strategies and initial power distribution network structure diagram. (a) The change of power supply recovery rate in three cases. (b) initial structure diagram of the distribution network.

The repair process is divided into several stages. The first stage is an emergency fault recovery plan, followed by multi-point fault repair until the fault is completely repaired and the power distribution network structure can operate normally. After simulation, the experimental results of the three schemes are shown in Table 9 The power supply recovery rates of the three schemes at each stage are illustrated in Fig 17a.

**Table 9:** Comparison of the results of the three cases.

Case	Loss of economic Benefits/kW h	Time required for complete recovery from failure/min	Total load Loss/kW
case 1	10522	485	5023
case 2	6524	478	5104
case 3	5339	505	3218

After the fault occurred, Islanding is divided, closed branches 8–21, 25–29, and 18–33, and branching branches 14–15 and 20–21 are reconstructed and restored. Currently, nodes 9, 10, 11, 12, and 22 are completely without power, the initial structure diagram of the distribution network is shown in Fig 17b.

**Fig 18:**
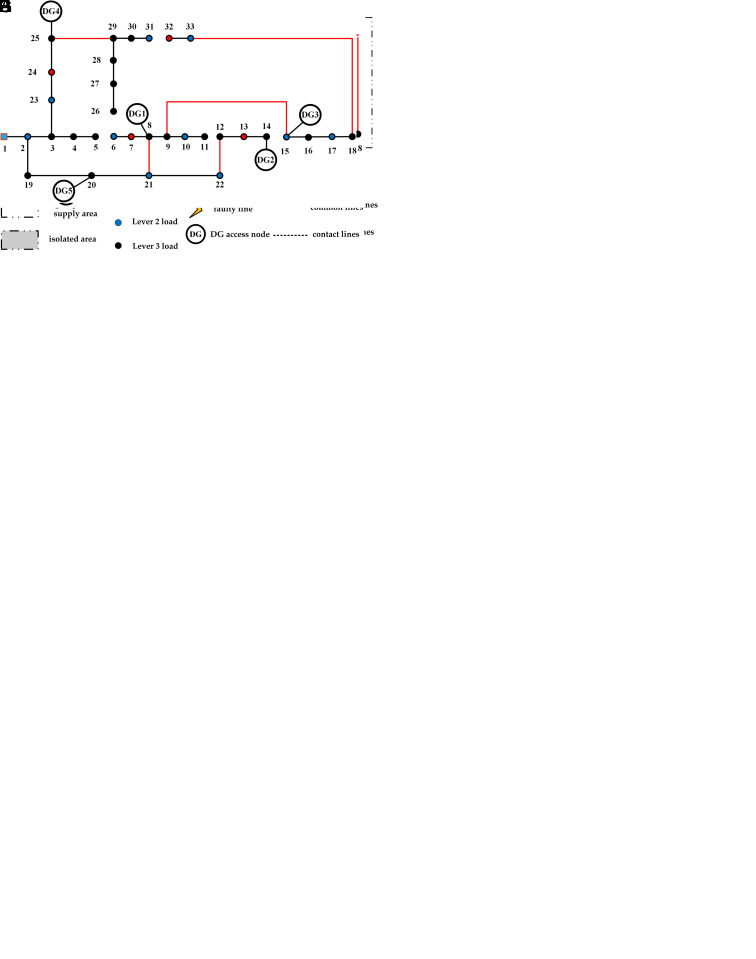
Process structure diagram of emergency repair strategies for two cases.

The fault repair and power distribution network reconfiguration processes in cases 2 and 3 are as follows:

In case 3: Repair the fault points C8 and C2 and close the branch circuits 32–33 and 24–25. Adjust the reconstruction recovery and fault repair plan, close the branch circuits 9–15 and 12–22, and disconnect the branch circuit 14–15. At this time, the load from the previous power outage has been slightly restored, and the structure of the distribution network in the second stage is shown in Fig 18a. Repair the fault points C4 and C5 and close the branches 26–27 and 6–7. Adjust the reconstruction recovery and repair plan, close the branches 15–16 and open the branches 27–28. At this point, all nodes have been significantly recovered, and the structure of the distribution network in the third stage is shown in Fig 18c. Repair the fault points C3 and C6 and close the branches 21–22 and 8–9. Adjust the reconstruction recovery and fault repair plan, close the branches 12–22, 14–15, and 27–28, and disconnect the branches 28–29 and 29–30. At this point, all nodes have been completely recovered, and the structure of the distribution network in the fourth stage is shown in Fig 18e. Repair the fault points C1 and C7 and close branches 2–19 and 12–13. Adjust the reconstruction recovery and fault repair plan, close branches 20–21, 29–30, and 12–22, and disconnect branches 5–6, 9–15, 10–11, and 30–31. At this point, all fault points have been repaired, the system has returned to normal operation, and the fault recovery process of the distribution network has been completed. The structure of the distribution network in the fifth stage is shown in Fig 18g.

In case 2: Repair the fault points C8 and C5 and close branches 32–33 and 6–7. Adjust the reconstruction recovery and repair plan, disconnect branches 7–8 and 30–31, and close branches 8–21 and 9–15. The structure of the distribution network in the second stage is shown in Fig 18b. Fault repair points C6 and C7 and close branches 8–9, 12–13, break off branches 9–10, 8–21, and close branches 7–8, 30–31. At this time, load recovery is extensive, and the structure of the distribution network in the third stage is shown in Fig 18d. Adjust the reconstruction, recovery and repair plan, repair the fault points C4 and C1 and close branches 26–27 and 2–19, close branches 8–21 and 9–10, and disconnect branches 4–5 and 27–28. At this point, all nodes have been completely recovered, and the structure of the distribution network in the fourth stage is shown in Fig 18e. Adjust the reconstruction, recovery and repair plan, repair fault points C2 and C3 and close branches 24–29 and 21–22, disconnect branches 5–6, 11–12 and 6–26, and close branches 4–5 and 27–28. At this point, all fault points have been repaired, the system has returned to normal operation, and the restoration process of the distribution network fault has been completed. The structure of the distribution network in the fifth stage is shown in Fig 18h.

#### 5.2.3. Comparative analysis.

**Fig 19:**
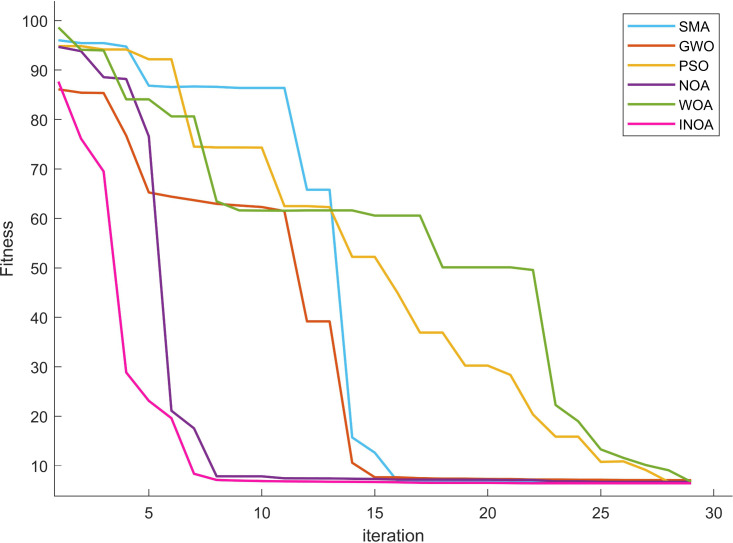
Comparison of convergence curves of different algorithms.

**Fig 20:**
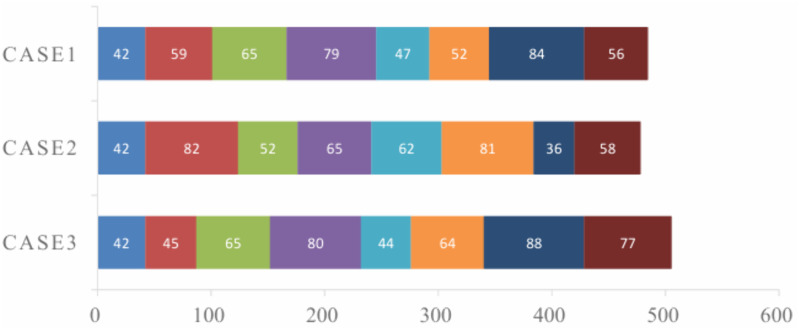
Comparison of Gantt charts for repair team scheduling under different cases.

To validate the effectiveness of the proposed INOA, it was compared with PSO, GWO, SMA, WOA, and NOA, which are commonly used to solve TSP problems. The number of iterations for each algorithm was set to 30, and the population size was set to 30. The change curves of their fitness values are shown in Fig 19. Gantt chart comparing repair team dispatching under different strategies, as shown in Fig 20.

As shown in Figure, after seven iterations, INOA converged to the optimal fitness value. Compared with other optimization algorithms, INOA converged faster and achieved better optimization results. While different cases result in varying repair dispatch plans, the repair times for the same fault points are relatively similar. The primary difference in total repair time is attributed to the travel speeds between the fault points.

After a multi-point failure, only the methods of islanding and distribution network restructuring cannot effectively recover the lost loads. Fault repair is very important for the faulty distribution network. The superiority of the cooperative strategy after a multi-point failure is analyzed by comparing the loss-benefit economics, the time taken to repair the failure, and the load-shedding amount at each node under the three cases.

During the emergency repairs in Case 1 and Case 2, varying priorities influenced the restoration strategies. In Case 2, which focused on the shortest repair path, the restoration of fault 5 was prioritized. Conversely, Case 3 aimed to minimize social losses by reducing load shedding, leading to a decision to prioritize the repair of node 2.

In Case 2, despite efforts to streamline the repair process, the main grid’s power supply range was not effectively expanded. As a result, the fault load continued to rely on the Distributed Generation (DG) system for power supply, failing to significantly reduce the overall power outage area. In contrast, after repairing fault 2 in Case 3, lines 24–25 were restored to normal operation, which substantially decreased the power outage area. This action effectively reduced the isolated island area of the DG system and improved the coordination between the main grid and the isolated island for restoration, thereby greatly enhancing power supply reliability. This improved coordination is the primary reason for the more rapid increase in load restoration rates observed in Cases 1 and 2 compared to other scenarios.

In phases 2–3, although Case 2’s emergency repair process succeeded in reducing the scope of the power outage, the time lost resulted in significant socio-economic losses due to the incomplete restoration of power to the affected loads. On the other hand, Case 3 effectively minimized the power outage area, allowing for network reconfiguration and the DG system to supply power to smaller load areas, thus maintaining a minimal level of service.

In the remaining phases, the main grid was able to utilize network reconfiguration to supply power effectively across the entire distribution network. However, this led to a slower rise or stabilization in the load recovery rate, as the focus shifted from emergency repairs to broader network reliability and stability.

A comparison of Case 1 and Case 2 shows that the load-cutting capacity of the two is roughly the same, and Case 2 reduces the socio-economic losses by 37.9% compared to Strategy 1. Since both strategies achieved a large-scale restoration of the power-off load in the third stage, but Case 2 had already achieved a full restoration of the distribution network system without load cutting in the subsequent fourth and fifth stages, improving system stability. Case 2 also reduces the repair time by 7 minutes compared to the random line repair, which reflects the speed of the shortest path strategy. The above results show that compared with the ordinary random path fault recovery and repair coordination strategy, the coordinated optimization strategy with the shortest path can significantly reduce the efficiency of fault recovery and speed up the restoration of load, thus achieving effective recovery of the power-off load.

Based on Case 2, Case 3, which is adopted in this paper, combines fault recovery with fault repair. The socio-economic loss is reduced by 17.2% compared with Case 2. Case 3 achieves a large-scale restoration of power failure loads in the second stage, and the load-shedding amount is reduced by 37% compared with Case 2, greatly reducing the phenomenon of load-shedding, improving the stability of the distribution network system, and further reducing social and economic losses. Strategy 3 compared to Case 2: Although the time consumed for fault repair has increased by 27 minutes, effective recovery of the load after a multi-point fault is ensured through reasonable reconstruction of the distribution network and islanding, which greatly reduces the economic losses caused by power outages. This can better improve user satisfaction and effectively maintain the stable operation of the power grid system.

## 6. Conclusions

This paper addresses the challenge of restoring distribution grids following fault events by proposing a coordinated optimization strategy for both fault restoration and emergency repairs. This approach aims to improve load restoration efficiency and minimize losses during system outages, ultimately enhancing the overall resilience of distribution grids. The key conclusions are as follows:

1) The proposed model thoroughly considers the coordination between fault recovery in distribution networks and emergency repairs. It includes dynamic isolation of faulted sections and real-time network reconfiguration based on different emergency repair strategies, optimizing the repair plan at each stage.
2) The integration of the INOA (Improved Network Optimization Algorithm) significantly enhances the efficiency of generating repair dispatch plans. Additionally, effective ring network coding strategies and variable discretization techniques are utilized to improve the solvability of the restoration problem.
3) The fault restoration strategy, which prioritizes maximizing load restoration as its optimization objective, effectively minimizes load shedding losses and accelerates the recovery of the distribution network’s performance. This is in contrast to coordination strategies that focus solely on shortest paths or random paths, thereby contributing to increased resilience in the distribution network.

Future research will focus on developing extreme weather scenarios and accounting for the uncertainties associated with renewable energy generation under such conditions. This initiative aims to align post-disaster recovery strategies more closely with real-world disaster situations. Additionally, efforts will be made to enhance the resilience of distribution grids in extreme conditions by addressing several key issues, including the pre-allocation of emergency repair resources, uncertainties during the repair processes, and the coordination of emergency power vehicles and multiple repair teams. Moreover, the performance of the INOA algorithm will be further refined and optimized to improve its effectiveness in these challenging scenarios.

## Nomenclature

**Table:** 

Parameters	
$X_{i,t}$	Load Power Outage Emergency Parameters
$c_{level}$	load classification system Parameters
$b_{i,t}$	different categories of load
$P_{W}$	light intensity during time
$P_{max}$	max limit of the power output capacity
A	area of the plate
η	efficiency of the battery plate
N	total set of nodes included in the distribution network
$\Delta F_{res}$	mount of system performance loss per unit time
$\delta_{i,t}$	power supply status of node *i* at time *t*
$J_{s,i}$	the time consumed by maintenance personnel to reach the fault point
$J_{y,i}$	time consumed to repair the fault line
M	total set of branches contained in the distribution network
$Z_{l}$	impedance on the *l*-th branch
$P_{l}^{2}$	active power flowing into the end of the *l*-th branch
$Q_{l}^{2}$	reactive power flowing into the end of the *l*-th branch
$\mu_{l,t}$	contact status of the *l*-th branch at time *t*
NF	set of sectional switches in the distribution network
NL	set of contact switches in the current distribution network
$y_{j}$	opening and closing status of the *j*-th switch
$N_{C}$	the set of failure points
$N_{h}$	set of nodes representing a power failure
$P_{t,i}$	the active power injected at node *i* at time *t*
$P_{t,DGi}$	the active power of the distributed generation at node *i*
$P_{t,Li}$	he active power of the load at node *i*
$Q_{t,i}$	the reactive power injected by the node *i* at time *t*
$Q_{t,DGi}$	the reactive power of the distributed generation at the node *i*
$Q_{t,Li}$	the reactive power of the load at the node *i*
$U_{t,i}$	the node voltages of nodes *i* at time *t*
$U_{t,j}$	the node voltages of nodes *j* at time *t*
$G_{ij}$	branch admittance of nodes *i* and *j* in the distribution network structure
$B_{ij}$	branch susceptance of nodes *i* and *j* in the distribution network structure
$\delta_{t,ij}$	power factor angle between nodes *i* and *j*
$I_{l}$	the current flowing on branch *l*
$I_{l,max}$	the upper limit of the current passing through branch *l*
$U_{i,min}$	the lower limit of voltage of node *i*
$U_{i,max}$	the upper limit of voltage of node *i*
$U_{i,t}$	the voltage at *t* time of node *i*
$P_{l}$	the power on branch *l*
$P_{l,m\alpha x}$	the upper limit of the power provided on branch *l*
Nk	total set of distribution network DG access nodes
$P_{a,t}^{DG}$	the active output of the a-th DG system at time *t*
${\hat{P}}_{a,t}^{DG}$	the redicted active output of the a-th DG system at time *t*
$Q_{a,t}^{DG}$	the reactive power output of the a-th DG at time *t*
$\theta_{a}^{DG}$	the capacity of the a-th DG at time *t*
$ESS_{a,t}^{DG}$	the DG active power output of the access node *i*
$P_{a,t}^{DG}$	the DG active power output of the access node *i*
$P_{L,i}$	the power required to load node *i*
g	the network topology of the distribution network after reconstruction
G	the set of all radial topologies in the distribution network
$cap_{sum}$	the total number of emergency repair resources owned by the overall maintenance members
$rep_{case(k)}$	all the emergency repair resources required to complete emergency repair case
case(k)	the sequence of fault repair points *k* to be repaired in an emergency repair case
$J_{y,k}$	the time consumed by maintenance staff in repairing the fault at point *k*
${\hat{J}}_{y,k}$	the estimated time consumed by maintenance staff *k* in repairing the fault
*K*	the total number of fault points
$\sum_{\forall k=K}road_{start}^{k}$	the maintenance members start the repair case from the starting point
$\sum_{\forall k=K}road_{k}^{final}$	the maintenance members end repair case final after the case fault -th*k*
$road_{start}^{k}$	The movement route from the starting point to the point for the maintenance staff to repair
